# Overcoming Basal Autophagy, Kangai Injection Enhances Cisplatin Cytotoxicity by Regulating FOXO3a-Dependent Autophagic Cell Death and Apoptosis in Human Lung Adenocarcinoma A549/DDP Cells

**DOI:** 10.1155/2022/6022981

**Published:** 2022-09-01

**Authors:** Huan Zhou, Pengyu Pan, Qiuyu Zhao, Wenjun Liu, Ye Sun, Jianbo Wang, Chunying Liu, Chun Wang

**Affiliations:** ^1^Department of Cell Biology, College of Integrated Chinese and Western Medicine, Liaoning University of Traditional Chinese Medicine, 79 Chongshan Eastern Road, Huanggu District, Shenyang 110847, China; ^2^Key Laboratory of Ministry of Education for Traditional Chinese Medicine (TCM) Viscera-State Theory and Applications, Liaoning University of Traditional Chinese Medicine, 79 Chongshan Eastern Road, Huanggu District, Shenyang 110847, China; ^3^Teaching and Experimental Center, Liaoning University of Traditional Chinese Medicine, 79 Chongshan Eastern Road, Huanggu District, Shenyang 110847, China; ^4^Key Laboratory of Environmental Pollution and Microecology of Liaoning Province, Shenyang Medical College, Shenyang 110034, China

## Abstract

Cisplatin resistance is one of the major obstacles in the treatment of nonsmall cell lung cancer (NSCLC). Kangai injection (KAI), a Chinese herbal medicine, has been used in tumors as adjuvant treatment, but its exact antitumor mechanism is still unclear. In this study, we first demonstrated that cisplatin-resistant A549/DDP cells showed a higher level of basal autophagy in response to cisplatin treatment with increasing autophagic protein expression levels of Beclin 1, p62, and LC3 compared to cisplatin-sensitive A549/DDP cells; then, we assessed the antitumor effect of KAI in cisplatin-resistant lung adenocarcinoma A549/DDP cells. Our results showed that KAI exhibited direct cytotoxic and chemosensitizing effects in A549/DDP cells. Combining KAI with cisplatin promoted A549/DDP cell apoptosis, which was confirmed by cell cycle arrest, condensed nuclear chromatin, annexin V fluorescein isothiocyanate/propidium iodide (Annexin V-FITC/PI) staining, and apoptosis-related protein expression. In addition, combining KAI with cisplatin induced autophagic cell death in A549/DDP cells with a high level of basal autophagy, as indicated by an increase in LC3 spot count, an accumulation of Beclin 1 and LC3 II, and reduced p62 protein expression. We also found that the apoptosis and autophagic cell death induced by cotreatment of KAI and cisplatin in A549/DDP cells were FOXO3a-dependent as indicated by decreased p-FOXO3a expression and increased FOXO3a nuclear localization, respectively. Furthermore, the *FOXO3a* gene knockdown assay further confirmed that KAI enhanced cisplatin cytotoxicity in A549/DDP cells with a high level of basal autophagy by inducing apoptosis and autophagic cell death in a FOXO3a-dependent manner. These findings suggest that the combination of KAI and cisplatin might support the potential clinical treatment as a novel strategy to overcome cisplatin resistance.

## 1. Introduction

cis-Diamine-dichloroplatinum (II) (best known as cisplatin) plays a crucial role in the treatment of a wide variety of cancers, especially advanced lung cancer. Nevertheless, long-term cisplatin treatment partially leads to acquiring resistance to cisplatin, which is the main cause of failure in chemotherapy. Several mechanisms are thought to be involved in cisplatin resistance, including a reduction in intracellular drug accumulation, an enhanced drug-detoxification system, increased DNA repair mechanisms, altered mitochondrial function, activated antiapoptotic defenses, and protective autophagy [1].

Autophagy is a cellular survival mechanism that is induced by intracellular and extracellular stresses. A growing number of studies have confirmed that autophagy promotes acquired chemoresistance in tumor cells in 5-Fu-resistant colorectal cancer cells (HCT8Fu), docetaxel-resistant lung cancer cells (A549/DTX), and cisplatin-resistant lung cancer cells (A549/DDP) [2–4]. However, chemoresistant cells, such as epirubicin-resistant breast cancer MDA-MB-231 cells or SUM159PT cells and adriamycin-resistant acute myeloid leukemia K562 cells, usually obtain a basal autophagic phenotype [5, 6]. As autophagy is a double-edged sword in the carcinogenic process, the question of how to overcome the basal autophagy in those chemoresistant cells, such as by inhibiting or activating autophagy, is worth considering in tumor treatment.

Autophagy, apoptosis, and other modes of cell death can control cell fate through cross-talk signals. Some transcription factors, such as Forkhead box O3a (FOXO3a), are considered to be cell-surveillance mechanisms designed to correct autophagy perturbations and confer apoptosis sensitization [7]. FOXO3a, the most important member of the Forkhead box O family, plays important roles in multiple biological processes, such as cell proliferation, differentiation, and apoptosis. Further, FOXO3a has been linked to chemoresistance, and sorafenib resistance via activation of the FOXO3a/autophagy signaling pathway in hepatocellular carcinoma cells [8, 9].

Traditional Chinese medicine (TCM) has attained great popularity in the alternative and complementary treatment of advanced cancers. The Kangai injection (KAI), consisting of ginseng, astragali radix, and kushen, has been widely applied in cancer treatment as an adjuvant method [10, 11]. More researches show that KAI in combination with chemotherapy has been widely used for treating NSCLC, colorectal cancer, breast cancer, etc. in clinical practice to improve the quality of patients' lives in China [12–14]. However, the underlying mechanisms of KAI at play in increasing chemotherapy sensitivity remain unknown. In this study, we evaluated the mechanism(s) of KAI's reversal of cisplatin resistance via a FOXO3a-mediated signaling pathway in lung adenocarcinoma A549/DDP cells.

## 2. Materials and Methods

### 2.1. Cell Line and Cell Culture

Cell lines of cisplatin-sensitive A549 cells and cisplatin-resistant A549/DDP cells were purchased from the Cancer Research Center of the Chinese Medical College. In McCoy's 5A medium (Genview, Houston, TX, USA), 1% penicillin streptomycin and 10% fetal bovine serum (Hyclone Laboratories, Logan, UT, USA) were added, and the cells were routinely cultured at 37°C in a humidified incubator containing 95% humidity and 5% CO_2_ at a low concentration of cisplatin (1 *μ*M) to maintain cisplatin resistance [15].

### 2.2. Reagents and Antibodies

KAI (note, a crude drug concentration of 400 mg/mL was used for the experiment) was obtained from Changbai Shan Pharmaceutical Co., Ltd. (Jilin, China), and cisplatin was purchased from Sigma-Aldrich (St. Louis, MO, USA). The required antibodies are shown in Table 1.

### 2.3. Cell Viability Assay

A549 or A549/DDP cells (5 × 10^3^ cells/well) of the logarithmic growth stage were subcultured into 96-well plates for 12 h and then treated with different concentrations of cisplatin (0, 1, 2, 4, 8, 16, 32, 64, and 128 *μ*M) for 24 h. The survival ability of cells was evaluated via a Cell Counting Kit-8 (CCK-8) (Beyotime, Shanghai, China) to obtain a cell growth curve and IC_50_ value for cisplatin. To determine the cell viability of A549/DDP cells exposed to KAI, cells were incubated with different concentrations of KAI (0, 20, 40, 80, and 160 mg/mL) for 26 h and then evaluated by CCK-8 assay to calculate the IC_5_, IC_10_, IC_20_, and IC_50_ values for KAI, respectively. In addition, for the assay of the cytotoxic effect of KAI with cisplatin, A549/DDP cells were first treated with various concentrations (20, 35, and 55 mg/mL) equivalent to the IC_5_, IC_10_, and IC_20_ values detected by the experiment of KAI mentioned above for 2 h and then coincubated with cisplatin for another 24 h. To confirm the mechanism of growth inhibition induced by coincubation with KAI and cisplatin, A549/DDP cells were pretreated with the caspase inhibitor z-Val-Ala-Asp-fluoromethyl ketone (z-VAD-fmk) (20 *μ*M) or autophagy inhibitor Bafilomycin A1 (10 nM) for 2 h; then, KAI and cisplatin were similarly processed. After incubating with the CCK-8 reagent, we measured the values of optical density (OD) at a wavelength of 450 nm using a microplate reader (Tecan Group, Männedorf, Switzerland).

### 2.4. Western Blot Analysis

After drug intervention or no drug intervention, A549 or A549/DDP cells were lysed and subjected to western blot analysis as a general method [16]. After treatment, the blots were probed with antibodies against Beclin 1, LC3, p62, cleaved caspase-3, p53, Bax, Bcl-2, FOXO3a, p-FOXO3a Ser253, p-FOXO3a Ser315, and p-FOXO3a Thr32; then, we detected the protein bands with ECL solution (Vazyme, Nanjing, China) using a Tanon 5200 imaging system (Tanon Science & Technology, Shanghai China) [17].

### 2.5. Flow Cytometry-Based Cell Cycle and Apoptosis Assay

After cotreatment with KAI and cisplatin, A549/DDP cells were harvested for cell cycle-distribution analysis by propidium iodide (PI) staining (Dingguo Changsheng Biotechnology, Beijing, China) and detected by flow cytometry (BD Biosciences, San Diego, CA, USA). For apoptosis detection by flow cytometry, A549/DDP cells were checked using an Annexin V-FITC/PI double staining kit (Vazyme, Nanjing, China). The results were analyzed using FlowJo software version 10.6.2 (BD Biosciences, San Diego, CA, USA) [18].

### 2.6. Cell Immunofluorescence Staining

Hoechst 33258 dye (Sigma-Aldrich, St. Louis, MO, USA) was used to observe the apoptosis characteristic. After cotreatment with KAI and cisplatin, A549/DDP cells were stained with 10 mg/mL of Hoechst 33258 staining reagent for 30 min. Then, we visualized the stained nuclei of A549/DDP cells under a fluorescence microscope (Leica, Wetzlar, Germany) [19]. A549/DDP cells were fixed with paraformaldehyde and permeabilized with methyl alcohol; inoculated by LC3 (1 : 200) or FOXO3a (1 : 200) antibodies, respectively; and then incubated with the secondary antibody (Alexa Fluor 594 goat antirabbit immunoglobulin G at 1 : 400; Proteintech, Rosemont, IL, China). With the 4′,6-diamidino-2-phenylindole (DAPI) strained for the nucleus, cells were captured by confocal microscopy (Olympus Corporation, Tokyo, Japan) or inverted fluorescence microscopy (Zeiss, Jena, Germany).

### 2.7. Quantitative Real-Time Polymerase Chain Reaction

Total RNA was extracted from A549/DDP cells using a TRIzol reagent (TransGen Biotech, Beijing, China). The synthesis of complementary DNA (cDNA) was reverse-transcribed using a cDNA synthesis kit (Cowin, Beijing, China). The mRNA expression levels of FOXO3a were calculated using the UltraSYBR mixture kit (Cowin, Beijing, China). *β*-Actin gene expression was chosen as an internal control. The adopted primer sequences are listed as follows: *FOXO3a* forward, 5′-TGACGACAGTCCCTCCC-3′ and reverse 3′-GCTGGCGTTAGAATTGGT-5′; *β-actin* forward, 5′-CACTGTGCCCATCTACGAGG-3′ and reverse 3′-TAATGTCACGCACGATTTCC-5′.

### 2.8. Small Interfering RNA (siRNA) Transfection

Cells were seeded in a 96-well plate with 5 × 10^3^ cells/well and cultured for 24 h. After incubation, siRNA-targeting FOXO3a (5′-GAGCTCTAGCTTCCCGTAT-3′) or siRNA-targeting control (RiboBio, Guangzhou, China) mixed with Golden Trans DR reagent (Golden Trans, Changchun, China) and added to basic McCoy's 5A medium according to the manufacturers' protocols.

### 2.9. Statistical Analysis

Each experiment was repeated in triplicate. All data are given as mean ± standard deviation (SD) values. The results were determined using SPSS version 19.0 (IBM Corporation, Armonk, NY, USA) and GraphPad Prism version 8.0 (GraphPad Software, San Diego, CA, USA). Two-tailed Student's *t*-tests were used to analyze differences between the two groups. An analysis of variance test was used to perform multiple comparisons of data. The survival curve was analyzed via the Kaplan-Meier method, and the curves were then compared using a log-rank test. *P* < 0.05 was regarded as a statistically significant threshold.

## 3. Results

### 3.1. Cisplatin-Resistant Lung Adenocarcinoma Cells Showed a Higher Level of Basal Autophagy in Response to Cisplatin Treatment

**Figure 1:**
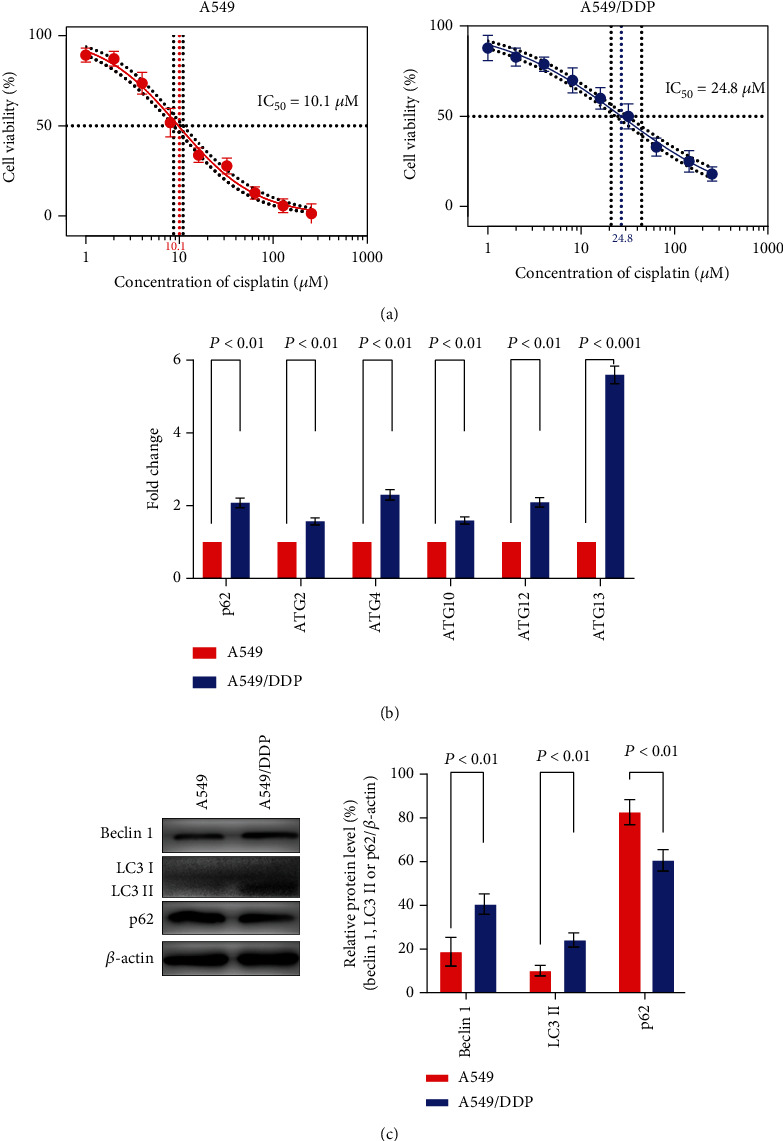
A549/DDP cells showed a higher level of basal autophagy in response to cisplatin treatment. (a) Direct cytotoxic effects of cisplatin in A549 and A549/DDP cells. A549 and A549/DDP cells were treated with various concentrations (0, 1, 2, 4, 8, 16, 32, 64, and 128 *μ*M) of cisplatin for 24 h. (b) RNA sequencing was used to compare the differentially expressed genes between A549 and A549/DDP cells. (c) Western blot analysis revealed the relative expression levels of autophagy-related proteins (Beclin 1, LC3 II, and p62) in A549 cells and A549/DDP cells. Every experiment result was repeated ≥3 times, and results are presented using mean ± SD values (*n* = 3).

To compare the sensitivity to cisplatin between the parental and cisplatin-resistant cells, the IC_50_ values of cisplatin in A549 cells and A549/DDP cells were detected by CCK-8 assay. As shown in Figure 1(a), the drug resistance of A549/DDP cells was significantly higher than that of A549 cells (24.8 *μ*M and 10.1 *μ*M, respectively; *P* < 0.05). The obtained IC_50_ value of A549 cells (~10 *μ*M) was used in the follow-up experiments.

We had compared the differentially expressed genes between the A549 and A549/DDP cells by RNA sequencing, and the results found that about 2200 differentially expressed mRNAs were upregulated in the A549/DDP cells, while the number of downregulated, differentially expressed mRNAs was approximately 4300 [20]. There were 29 autophagy-related genes which were upregulated at the transcription levels in A549/DDP cells compared to in A549 cells, among which 7 genes had statistically significant differences (Figure 1(b)). Then, we analyzed some protein-expression levels in A549 and A549/DDP cells. The results showed that Beclin 1 and LC3 II, playing key roles in the initiation of autophagy, were upregulated in A549/DDP cells; meanwhile, p62, an autophagy adaptor protein facilitating selective degradation of protein cargo via an autophagy-activation pathway, was decreased in A549/DDP cells compared to A549 cells (*P* < 0.01) (Figure 1(c)).

### 3.2. KAI Enhances Cisplatin Cytotoxicity in A549/DDP Cells

**Figure 2:**
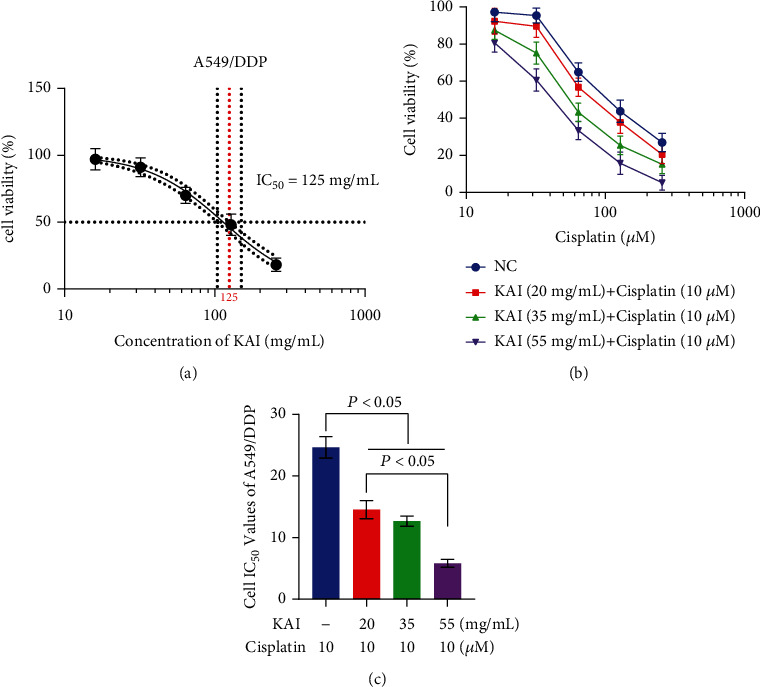
KAI improves cisplatin resistance of A549/DDP cells. (a) A549/DDP cell-inhibition rate curves with different concentrations (0, 20, 40, 80, and 160 mg/mL) of KAI administered for 24 h. (b) KAI at concentrations of IC_5_, IC_10_, and IC_20_ combined with cisplatin were used to determine the cytotoxicity of A549/DDP cells. (c) The IC_50_ value of A549/DDP cells was detected by cisplatin combined with KAI at IC_5_, IC_10_, and IC_20_ concentrations. Every experiment result was repeated ≥3 times, and results are presented using mean ± SD values (*n* = 3).

KAI exhibited a direct anticancer effect on A549/DDP cells with IC_5_, IC_10_, IC_20_, and IC_50_ values of 20, 35, 55, and 125 mg/mL, respectively (Figure 2(a)). Meanwhile, different concentrations of KAI combined with 10 *μ*M of cisplatin were used to treat A549/DDP cells. With an increase in the KAI concentration, the survival rate of A549/DDP cells decreased significantly (Figure 2(b)). In addition, when A549/DDP cells were cotreated with KAI (20, 35, or 55 mg/mL) and cisplatin (10 *μ*M), the IC_50_ value of cisplatin decreased from 24.8 *μ*M to 14.5 *μ*M, 12.6 *μ*M, and 5.9 *μ*M, respectively (Figure 2(c)).

### 3.3. Combination of KAI- and Cisplatin-Induced Apoptosis in A549/DDP Cells

**Figure 3:**
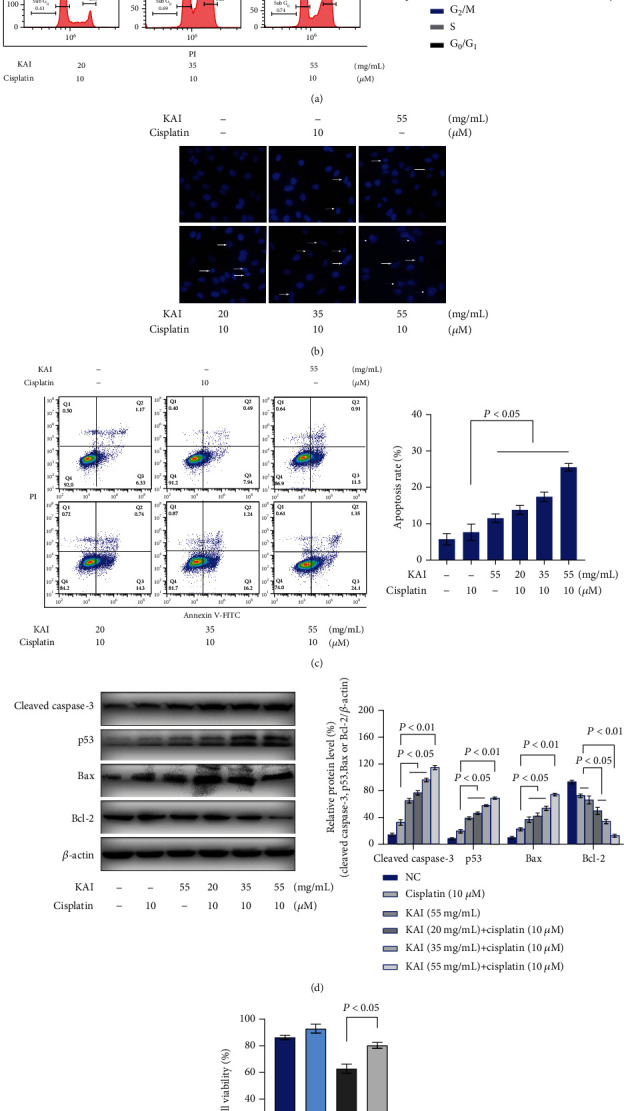
A combination of KAI and cisplatin promoted apoptosis in cisplatin-resistant A549/DDP cells. (a) A549/DDP cells were treated with KAI for 2 h; then, cisplatin (10 *μ*M) was added for combined treatment for another 24 h. The effects of cotreatment with KAI and cisplatin on the cell cycle of A549/DDP cells were detected by flow cytometry. (b) The nuclear chromatin condensation of A549/DDP cells was observed by fluorescence microscopy following staining with Hoechst 33258. (c) Annexin V-FITC/PI double staining of A549/DDP cells was performed to detect apoptosis by flow cytometry. (d) Western blot analysis showed apoptosis-related proteins (cleaved caspase-3, p53, Bax, and Bcl-2). *β*-Actin was used as a loading control. (e) A549/DDP cells were treated with 20 *μ*M of z-Val-Ala-Asp-fluoromethyl ketone for 24 h, then cotreated with KAI and cisplatin. Every experiment result was repeated ≥3 times, and results are presented using mean ± SD values (*n* = 3).

To investigate the effects of cotreatment with KAI and cisplatin on the cell cycle and apoptosis, A549/DDP cells were dyed with PI and analyzed by flow cytometry. A significant increase in the proportion of cells in the G_2_/M phase was seen as the KAI concentration increased, reflecting cell cycle arrest in this stage (Figure 3(a)). Significant apoptosis was triggered by cotreatment with KAI and cisplatin as observed by fluorescence microscopy with Hoechst 33258 dye and flow cytometry with Annexin V-FITC/PI double staining (Figures 3(b) and 3(c)). Furthermore, western blot analysis showed that the expression levels of proapoptotic proteins (cleaved caspase-3 and Bax) increased and the expression levels of antiapoptotic proteins (Bcl-2) decreased. Additionally, the amount of p53 protein, which participates in mitochondria-associated apoptosis, increased gradually with KAI and cisplatin cotreatment (Figure 3(d)). Nevertheless, apoptosis inhibitors could partly block the cell death induced by cotreatment with KAI and cisplatin, which means that another mode of death may be present in the cell death induced by KAI and cisplatin (Figure 3(e)).

### 3.4. Combination of KAI- and Cisplatin-Induced Autophagic Cell Death in A549/DDP Cells

**Figure 4:**
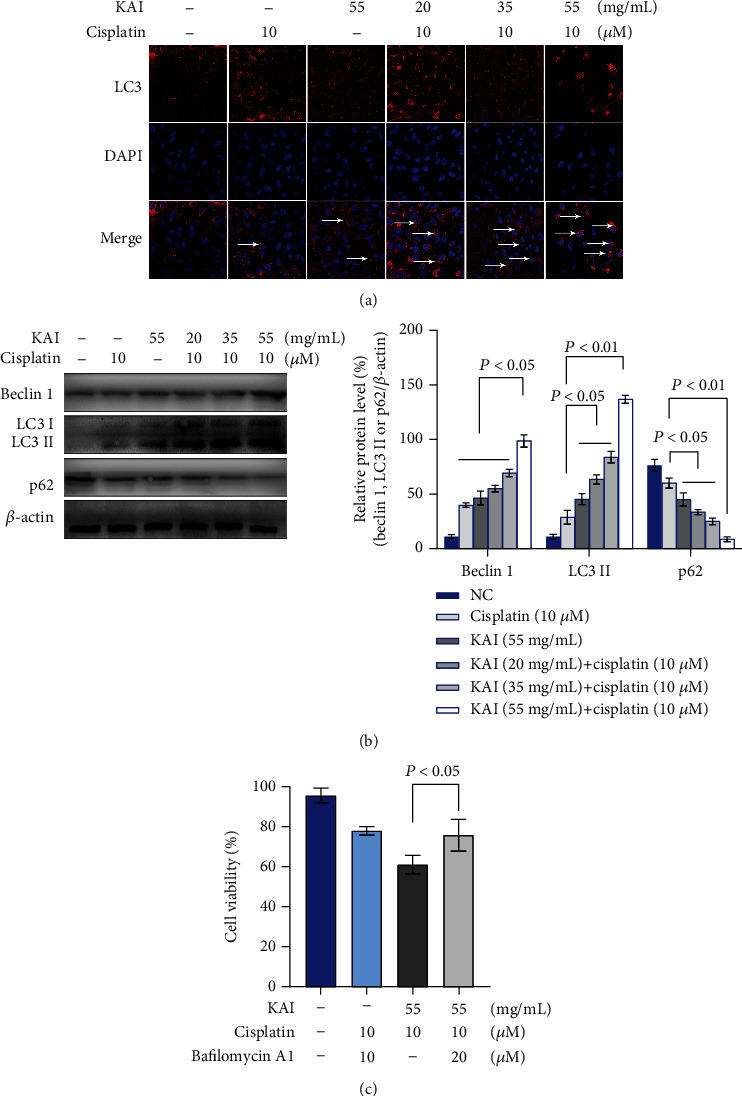
Combination of KAI- and cisplatin-triggered autophagy induction in A549/DDP cells. (a) A549/DDP cells were treated with KAI for 2 h; then, cisplatin (10 *μ*M) was added for combined treatment for another 24 h. Staining with LC3 autophagy immunofluorescence and DAPI in A549/DDP cells, observed by confocal microscopy. Red: LC3; blue: DAPI. (b) Western blot showed autophagy-related proteins (Beclin 1, LC3 II, and p62). *β*-Actin was the loading control. (c) A549/DDP cells were treated with 10 nM of Bafilomycin A1 for 24 h and then cotreated with KAI and cisplatin for 24 h. Every experiment result was repeated ≥3 times, and results are presented using mean ± SD values (*n* = 3).

As shown in Figure 4(a), cotreatment with KAI and cisplatin significantly enhanced the autophagy of A549/DDP cells, with the fluorescence spots of autophagy LC3 gradually increasing in number. The increasing autophagy was further proven by western blot analysis, which revealed that the expression levels of Beclin 1 and LC3 II were gradually rising, while that of p62 was gradually decreasing (Figure 4(b)). More importantly, the autophagy inhibitors could only block part of the cell death induced by cotreatment with KAI and cisplatin, which confirmed that autophagic cell death may participate in the cell death induced by KAI and cisplatin (Figure 4(c)).

### 3.5. FOXO3a Is Involved in the Apoptosis and Autophagic Cell Death Induced by the Combination of KAI and Cisplatin in A549/DDP Cells

**Figure 5:**
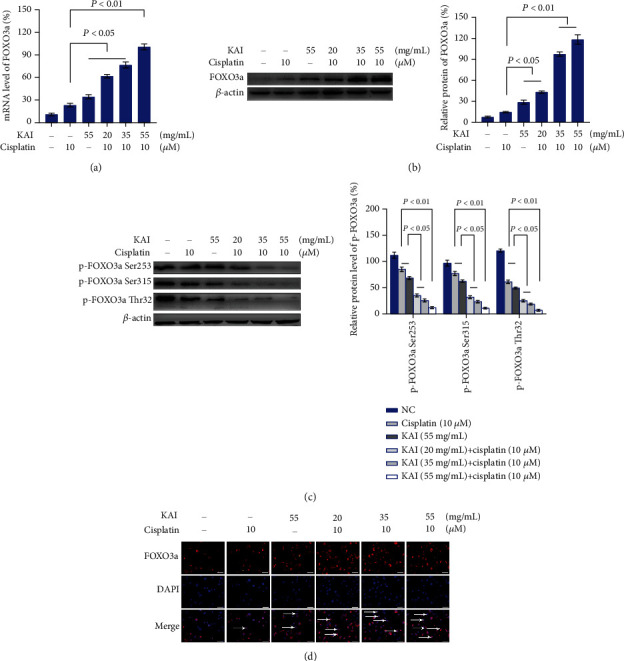
The combination of KAI and cisplatin in A549/DDP cells enhanced apoptosis and autophagy via FOXO3a. (a) A549/DDP cells were treated with KAI for 2 h, and then, cisplatin (10 *μ*M) was added for combined treatment for another 24 h. Real-time polymerase chain reaction and western blot (b) analysis of the expression of FOXO3a in A549/DDP cells. (c) Western blot analysis of the protein expression levels of the FOXO3a phosphorylation sites of Thr32, Ser253, and Ser315. *β*-Actin was used as a loading control. (d) Immunofluorescence antibodies and DAPI were double-stained to observe the nuclear localization of FOXO3a. Red: FOXO3a; blue: DAPI. Every experiment result was repeated ≥3 times, and results are presented using mean ± SD values (*n* = 3).

Transcription factor FOXO3a plays a crucial role in autophagy and apoptosis. Here, we tried to find whether FOXO3a is involved in the apoptosis and autophagic cell death of A549/DDP cells. We analyzed the mRNA and protein expression levels of FOXO3a, which were all upregulated relative to those achieved with the cisplatin-alone intervention (Figures 5(a) and 5(b)). Moreover, cotreatment with KAI and cisplatin could effectively reduce the expression of phosphorylated FOXO3a proteins at the Thr32, Ser253, and Ser315 sites and increase the level of nuclear localization (Figures 5(c) and 5(d)).

**Figure 6:**
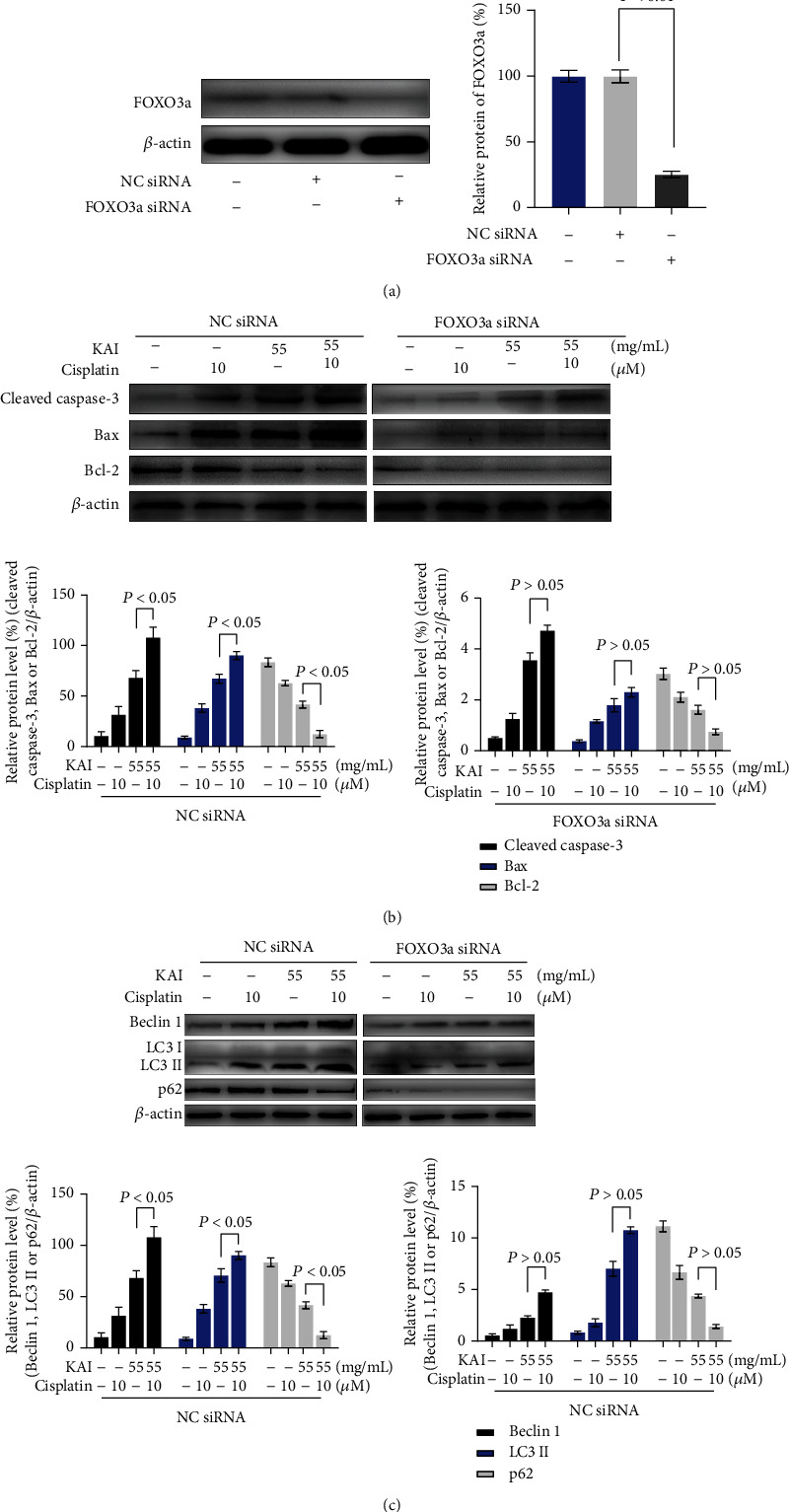
(a) Gene silencing of *FOXO3a* was examined via western blot analysis in A549/DDP cells, which revealed that silencing the *FOXO3a* gene inhibited apoptosis (b) and autophagy (c) through a combination of KAI and cisplatin treatment of A549/DDP cells. Every experiment result was repeated ≥3 times, and results are presented using mean ± SD values (*n* = 3).

To further confirm that the transcription factor FOXO3a participates in the apoptosis and autophagic cell death induced by cotreatment with KAI and cisplatin, we knocked down the *FOXO3a* gene in A549/DDP cells (Figure 6(a)). Knocked down *FOXO3a* gene could inhibit the apoptosis and autophagy activated by the combination of KAI and cisplatin (Figures 6(b) and 6(c)).

## 4. Discussion

Autophagy is the mechanism by which proteins, bulk cytoplasm, and damaged organelles are incorporated into double-membrane intracellular vesicles to be degraded through the machinery of lysosomes. Autophagy helps cancer cells maintain cellular homeostasis under microenvironmental stresses, such as starvation, hypoxia, or chemotherapies. Some studies have shown that increased basal autophagy can usually be detected in those resistant cancer cells compared to parental cells [5, 6]. In our previous study, we compared the mRNA transcript between the cisplatin-resistant A549/DDP cells and the parental A549 cells. Compared to A549 cells, the number of upregulated, differentially expressed mRNAs was approximately 2200, which included autophagy-related genes, such as *p62*, *ATG2*, *ATG4*, *ATG10*, *ATG12*, and *ATG13* [20]. In the current study, we also observed that Beclin 1 and LC3 II proteins were upregulated in cisplatin-resistant A549/DDP cells compared to parental A549 cells by a western blot method. Meanwhile, p62, an autophagy adaptor protein that facilitates selective degradation of protein cargo via an autophagy-activation pathway, was decreased in A549/DDP cells. These results suggest that cisplatin-resistant lung adenocarcinoma cells obtained phenotype resistance to cisplatin in response to greater basal autophagy.

Nevertheless, the mechanism underlying the switch from protective autophagy to autophagic cell death is still not completely clear. Apoptosis and autophagy, two important intracellular processes that maintain organism homeostasis and promote survival, are mutually independent but interrelated [21, 22]. Apoptosis sensitization is increased by autophagy inhibition or by promoting autophagic cell death and apoptosis by the autophagy activator through a cross-talk between apoptosis and autophagy, which are all effective methods for treating tumors. TCM, as an adjuvant cancer treatment, plays important roles in almost all cancers, including by suppressing tumor progression, relieving the discomfort of chemotherapy or radiotherapy, improving immune system function, and increasing the sensitivity of chemotherapeutics and radiotherapeutics. We have disclosed that the mechanism of Bu-Zhong-Yi-Qi decoction, the water extract of a TCM formulation, can enhance cisplatin cytotoxicity in A549/DDP cells through the induction of apoptosis and autophagic cell death [18]. In this study, we first showed that KAI could sensitize cisplatin to induce apoptosis by cell cycle arrest in the G_2_/M phase in A549/DDP cells, enhancing apoptosis cascade cleavage of cleaved caspase-3 and the change in the Bcl-2 family of proteins. The p53 protein, described as the “guardian of the genome” that interacts with Bcl-2 and Bcl-xL on the mitochondrial outer membrane to induce mitochondria-associated apoptosis, was detected to increase gradually with cisplatin and Shenqi Fuzheng Injection (a traditional Chinese herbal injection) cotreatment [23]. Next, KAI combined with cisplatin also induced autophagic cell death by enhancing autophagy degradation, thereby enhancing the toxic effect of cisplatin on cisplatin-resistant A549/DDP cells. Previous studies have revealed the antitumor mechanism of KAI that inhibits gastric cancer cell proliferation through the interleukin-6/STAT3 pathway [24]. The multiple antitumor pharmacological effects known to exist might originate from the active compounds of KAI, Radix ginseng rubra, and Radix ophiopogonis, which induce tumor cell apoptosis and inhibit tumor cell proliferation, invasion, and metastasis, helping to increase the sensitivity of chemotherapy [11].

Transcription factor FOXO3a plays a crucial role in autophagy and apoptosis [25]. Posttranslational modifications, including phosphorylation, acetylation, ubiquitination, and methylation, are fundamental processes for the realization of FOXO3a functions that cause changes in subcellular location, molecular half-life, DNA-binding affinity, and/or interaction with other cellular proteins [26]. In the cell nucleus, FOXO3a exhibits its transcription factor activity by recognizing and binding to target gene promoters and inducing the transcription of target genes. Some autophagy-related genes, such *LC3*, *Beclin 1*, and *Atg12*, and apoptosis-related genes, including *Bim* and *FasL*, are all regulated by FOXO3a [27]. A series of kinases phosphorylate the FOXO3a protein at Thr32, Ser253, Ser315, and Ser644, exporting FOXO3a from the cell nucleus to the cytoplasm. After the cytoplasmic retention, FOXO3a is ubiquitinated and then degraded by proteasome [26]. We observed that the expression levels of FOXO3a mRNA and protein were upregulated by a combination of KAI and cisplatin in cisplatin-resistant A549/DDP cells. Besides, the phosphorylated FOXO3a at Thr32, Ser253, and Ser315 were all inhibited apparently with an increasing concentration of KAI combined with cisplatin. More importantly, knocked down *FOXO3a* gene could inhibit the phenomena of upregulated autophagy and activated apoptosis induced by the combination of KAI and cisplatin. Recent studies have revealed that FOXO3 is at the center of a homeostatic feedback loop to regulate autophagy, and FOXO3 plays an important role in correcting autophagy inhibition and conferring apoptosis sensitization by the ability of FOXO3 to transactivate the proapoptotic gene BBC3/PUMA [28]. From the results above, we infer that combination of KAI and cisplatin can overcome basal autophagy and induce autophagic cell death and apoptosis in cisplatin-resistant A549/DDP cells by upregulating FOXO3a expression and elevating its activity.

**Figure 7:**
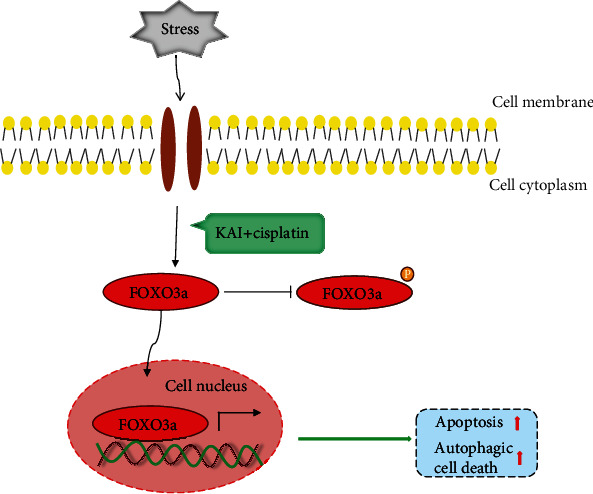
Combination of KAI- and cisplatin-mediated apoptosis and autophagic cell death though FOXO3a.

Although a high level of basal autophagy is a major obstacle in chemointervention, the signaling pathway of the FOXO3a-dependent autophagy and apoptosis may be a critical target in cancer treatment. In this context, the combination of KAI and cisplatin could help to overcome the basal autophagy in cisplatin-resistant lung adenocarcinoma cells and induce autophagic cell death and apoptosis by upregulating FOXO3a expression, elevating its activity, and promoting its nucleus location (Figure 7). These findings highlight that TCMs could be used as potential chemotherapy sensitizers in cancer adjuvant therapy in clinical practice.

## Figures and Tables

**Table 1:** Antibodies used in the experiment.

Antibody name	Firm	Host	Dilution
Beclin 1	Proteintech	Rabbit	1 : 500
LC3	Abcam	Rabbit	1 : 500
p62	Proteintech	Rabbit	1 : 1000
Bax	Cell Signaling Technology	Rabbit	1 : 1000
Bcl-2	Cell Signaling Technology	Rabbit	1 : 1000
p53	Proteintech	Rabbit	1 : 500
Cleaved caspase-3	Cell Signaling Technology	Rabbit	1 : 1000
FOXO3a	Cell Signaling Technology	Rabbit	1 : 1000
p-FOXO3a Ser315	Cell Signaling Technology	Rabbit	1 : 1000
p-FOXO3a Ser253	Cell Signaling Technology	Rabbit	1 : 1000
p-FOXO3a Thr32	Cell Signaling Technology	Rabbit	1 : 1000
*β*-Actin	Cell Signaling Technology	Rabbit	1 : 1000

## Data Availability

The research data are reflected in the article, and the initial data are available from the corresponding author upon request.
